# Improvement of the production of bio-oil and biodiesel from Egyptian Jatropha seeds by using microwave and ultrasonic

**DOI:** 10.1038/s41598-024-51579-6

**Published:** 2024-01-22

**Authors:** El-Sayed G. Khater, Soha A. AbdAlla, Adel H. Bahnasawy, Hassan M. AbuHashish

**Affiliations:** 1 Agricultural and Biosystems Engineering Department, Faculty of Agriculture, Benha University, Moshtohor, P.O. Box 13736, Toukh, Kalubia Egypt; 2 Mechanical Engineering Department, Engineering Research Division, National Research Centre, Giza, Egypt

**Keywords:** Ecology, Environmental sciences, Engineering

## Abstract

Bio-diesel is used for engine as a replacement of diesel fuel which is characterized by lower emission, low pollution and renewable some of fuel. This study focus on how to enhance the production of bio-oil from Jatropha seeds by using microwave and ultrasonic as pre-treatments. To achieve that, the effects of extraction temperature (60, 80, 100 and 120 °C) and extraction screw speed (60, 90 and 120 rpm) on oil extraction yield and quality, extraction energy requirements and extraction time and were studied. Studying the effect of pretreatments by microwave and ultrasonic on the yield, energy and time of extraction were studied. The results most important indicate that the highest oil yield (25.1%) was recorded at 120 °C extraction temperature and 60 rpm screw speed. The energy required for extraction ranged from 8 to 11.5 W.h depending on temperature and speed of extraction. The results indicated that using both pretreatments improve the oil yield by 5.03% for microwave and by 6.75% for ultrasonic. Finally, the results concluded that to produce 1 kg of biodiesel you need 1.1 kg raw oil and consume from 2052.5 W.h energy requirement.

## Introduction

Biomass is one of the renewable energy sources. Biomass is an organic material that comes from living organisms, such as plants and animals. The most common biomass materials used for energy are plants, wood, and waste products. The energy from these organisms can be transformed into usable energy through direct and indirect means. Biomass can be burned to create heat (direct), or processed into biofuel (indirect). Different types of energy are created through several ways such as: direct firing, co-firing, pyrolysis, gasification, and anaerobic decomposition. All these ways involve thermal conversion. Thermal conversion involves heating the biomass feedstock in order to burn, dehydrate, or stabilize it. The most familiar biomass feed stocks for thermal conversion are raw materials such as municipal solid waste (MSW) and scraps from paper or lumber mills. Before burning the biomass, it must be dried^1^.

Biodiesel is a liquid obtained from chemical process from vegetable oils or animal fats and can be used in diesel engines alone or mixed with diesel oil. Biodiesel has many advantages as a replacement for diesel fuel which include renewable, low toxicity, degradable, lower emission, lower health risk, has no sulfur dioxide and higher in flash point (100 °C minimum). The disadvantages of biodiesel are lower calorific value compared to diesel fuel, higher in NO_2_ emission, higher freezing point, less stable, can cause problem in the valves and injection system^2^. Biodiesel production is the process bio-fuel through chemical reaction. It is chemically known as fatty acid methyl ester (FAME)^3^. Diesel fuel is the most consumed fuel to fossil fuel in the world compared to other types of fuel^4^. Biodiesel is an important alternative fuel to fossil fuel^5^. It is characterized by biodegradable, non-toxic and free from sulfur^6^. It provide excellent engine lubrication and better combustion, low emissions, due to the existence of oxygen in the esters, complete combustion occurs in biodiesel compared to conventional diesel^7,8^.

Biodiesel could be rapeseed oil, palm oil, coconut oil and sunflower oil or nonedible oils such as Jatropha, castor, neem and karanja^9^. Low cost is the main factor for the profitability and sustainability of biodiesel business, because it is lower in prices, as its cost of biodiesel production depends on the cost of crude oil (about 75–90% of the cost is paid for obtaining crude oil)^10^.

Jatropha is planted in Egypt recently and used the wastewater because they are drought tolerance, rapid growth, and easy propagation, higher oil content than other oil crops and it has oil content of 30–50%^11^. The cultivated area of Jatropha around 900,000 ha around the world, 760,000 in Asia, 120,000 in Africa and perhaps 20,000 in Latin America^12^. All desert areas of Upper Egypt governorates and in the New Valley are considered potentially proper for Jatropha plantations. Such marginal land which has been planted with Jatropha in Egypt presently covers 844 hectares^13^.

Jatropha oil is an important source for biodiesel production in Egypt as sustainable product due to it can be grown in different conditions and is renewable oil that is not competitive with the food market^14^. In addition to, the alkyl ester of JCO meets biodiesel standards in many countries^15^.

Jatropha oil could be extracted by several methods, namely solvent extraction and mechanical pressure extraction. Solvent extraction method is considered one of the most efficient methods for extracting vegetable oils with an extraction rate of Jatropha oil reaches 99%. Despite of it is an effective and consistent method, it is the highest in cost, time, energy and consumption, and the least safe for the use of dangerous chemicals. Therefore, using mechanical pressure in extraction is preferable which Jatropha oil reaches 85%^16^.

Mechanical extraction is conducted by cylinder hole type screw presses or filters, which can be fed whole or ground seeds, with or without heat treatment. Grinding seeds could improve oil extraction because increases contact area, opening of pores, breakage of cell walls, reduction of oil viscosity and moisture^10^. The pretreatment unit alone decreases an amount of energy in Jatropha seeds before actual oil extraction which helps in oil extraction in Jatropha seeds by 24% of the total internal energy of solvent extraction processes and 66% for mechanical extraction. Screw press type is the most common type used in mechanical extraction which yields an extraction between 65 and 90% by weight and when pre-treated, the efficiency of Jatropha seed oil is 96%^17^.

Microwave pretreatments of oil extraction enhance the oil yield and quality with lower energy consumption, safer time and lower solvent levels compared to traditional methods^18^. Oil treated with microwave has the same properties of conventionally extracted oil such as acidity value, peroxide value and oil composition^19^. Microwave radiation causes fraction which leads to heating. Fats have low specific heat which cause vulnerable to this radiation and makes permanent pores in seeds resulting in higher yields and improved quality. It is considered as an alternative to traditional method with quicker processing, lower energy consumption and shorter exposure time^20,21^.

Ultrasonic is a new innovative technology that utilizes radiant waves at the rate of 18–20 kHz^22^. These waves causes vibration and heat, which destruct the membrane of the plant cell walls and cause them thinner and facilitating the release of the contents and enhancing the mass transfer of the solvent from the solution to the particles, thus increasing the yield of the extracted oil and improving the contact between solvents and plant materials^23^. The advantage of using ultrasonic method in extraction that it improves the efficiency of oil extraction, the quality of the extracted oil, the content of phenolic compounds and the safety of the process being environmentally friendly, safer, lowering extraction time and decreasing energy consumption.

Using the traditional methods of oil extraction result in low oil yield, low extraction efficiency, high energy consumption and low quality of oil extracted, therefore, using pretreatments of seeds was the main idea to improve oil yield and quality as well as reduce energy consumption. Microwave and ultrasonic pretreatments are the most commonly used pretreatments in oil extraction for seeds. The main aim of this work is to enhance the biodiesel production from Jatropha seeds oil.

## Experimental procedures

The main experiment was carried out in National Research Centre, Giza, Egypt. During summer season of 2022.

### Egyptian Jatropha seeds

**Figure 1:**
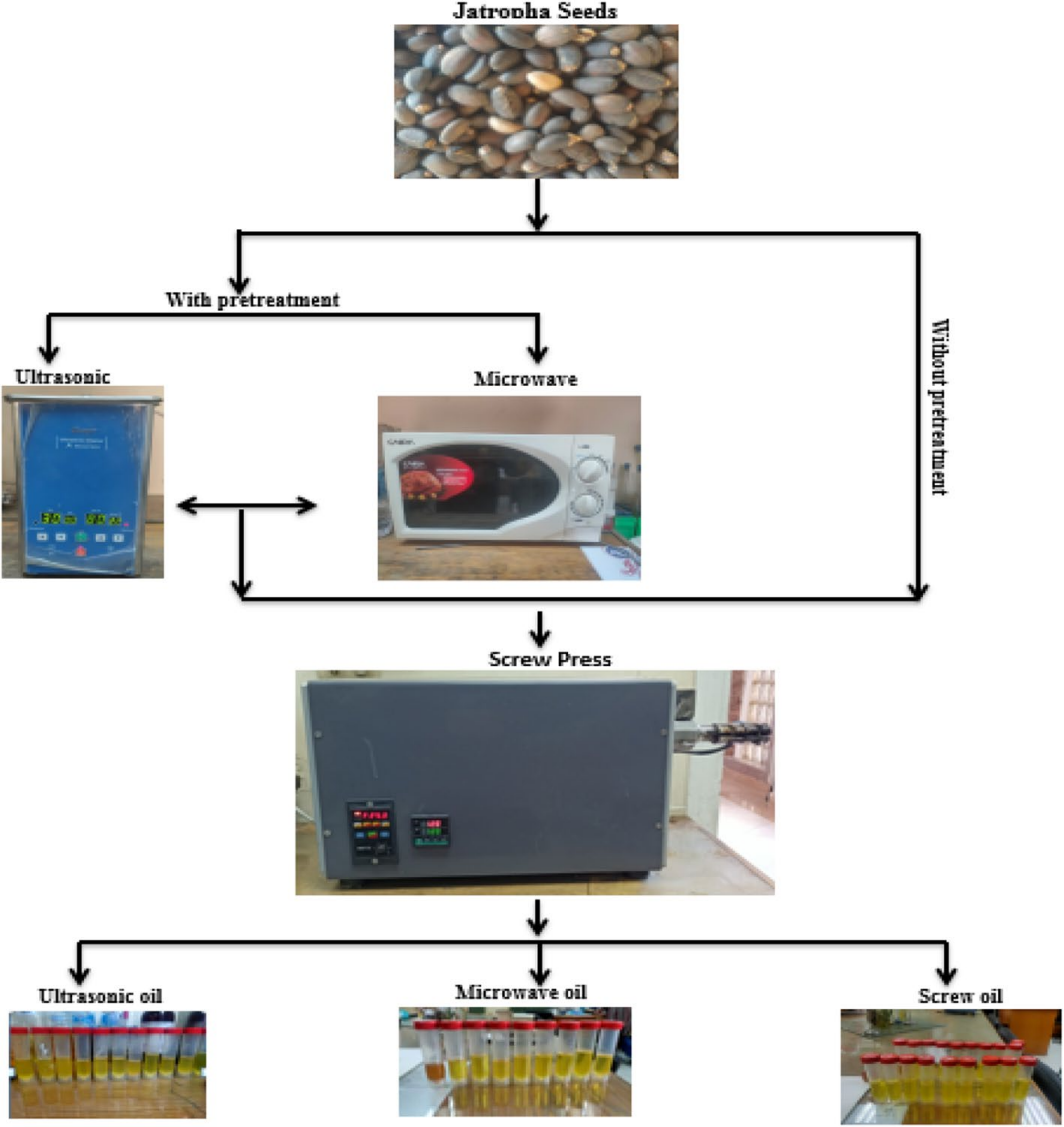
Schematic diagram of oil extraction by screw press from Egyptian Jatropha seeds.

Jatropha seeds were obtained from a privet farm in Al-Gabal Al-Asfar, Cairo. Castor seeds have been used under the permission of Benha University regulations. The seeds were peeled manually and crushed to ensure that the seeds contained the seed embryo that contains the oil content and that the seeds were not empty so that the empty seed husks would not absorb a percentage of the oil. The bio-oil was extracted from the Egyptian castor seeds by a screw press. Seeds were pre-treatment with microwave and ultrasonic methods compared to untreated seeds to enhance the production of bio-oil from castor seeds. The schematic diagram in Fig. 1 shows the sequence of oil extraction by screw press from castor seeds.

### Mechanical screw extraction method

**Figure 2:**
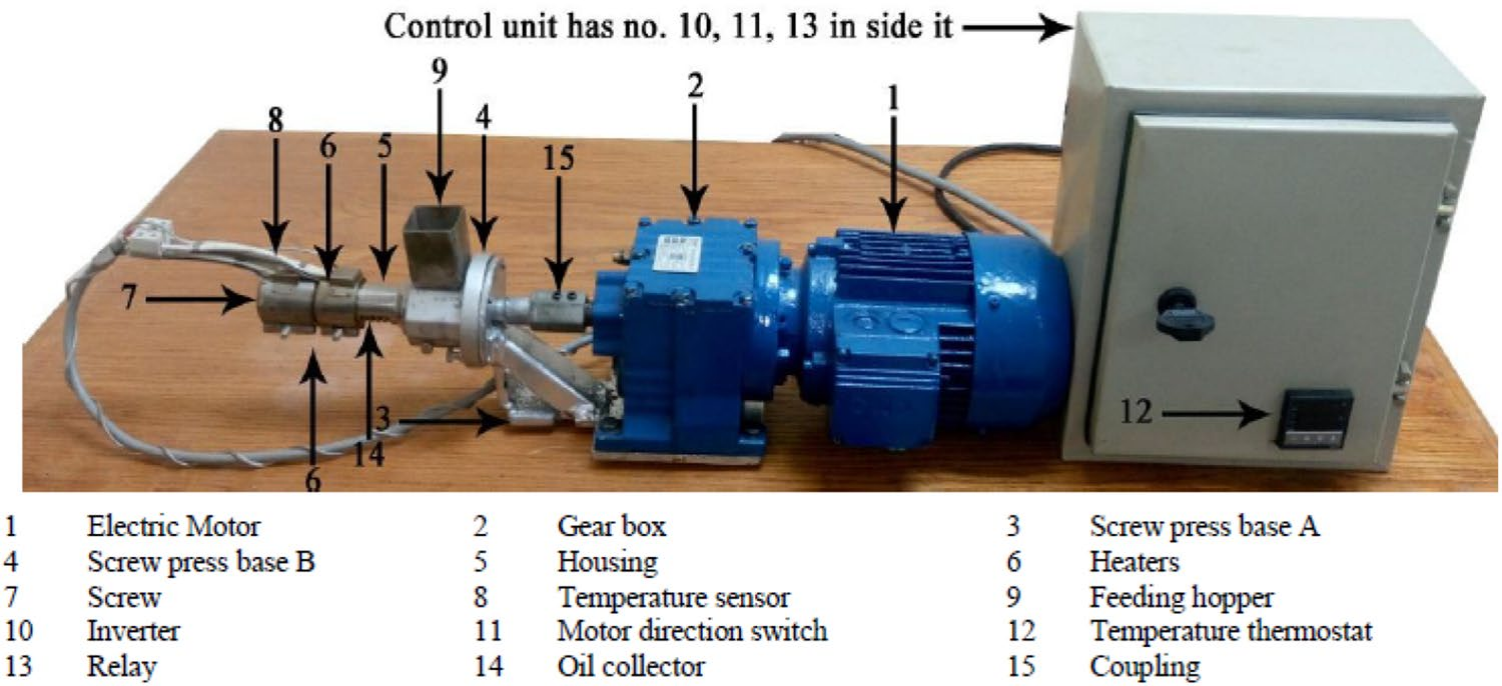
Photograph of the screw press.

The oil extraction was performed using a specially designed laboratory scale mechanical screw press located in the National Research Center, Giza, Egypt. The specifications of the device are quoted from Ibrahim et al.^15^. A photograph of the screw press and its are shown in Fig. 2.

Jatropha seeds were pressed at three speeds of 60, 90 and 120 rpm and four extraction temperatures of 60, 80, 100 and 120 °C. The oil yield weight and energy were measured for each treatment by energy meter. Also, the time required for pressing for each treatment was recorded. Three replicates were made and average was taken**.**

### Pretreatments

#### Microwave pretreatment

A microwave with power 700 W and 20 L capacity and made of Korea, CARIA model was used. The CARIA is owned by the Benha University and this work is done under the rules of this university. The treatments were selected according to Ref.^22^. The Jatropha seeds were heated in the microwave and the samples were weighed before and after heating to measure the loss in moisture. The samples were heated in the microwave for 1, 3 and 5 min at three levels power low, medium and high and then the samples were pressed in the screw press immediately after heating in the microwave at the optimum parameters that were determined in the screw press. The energy required for heating in the microwave and the power for each samples were measured by energy meter and then both the oil yield was recorded.

#### Ultrasonic pretreatment

Ultrasonic device (Model: UD50Sh-2.5LQ, Ultrasonic power: 50 W, Total consumption power: 160 W and Power supply: 220 VAC, 50 Hz) and made of USA, Eumax model was used. The Eumax is owned by the Benha University and this work is done under the rules of this university. Three different exposure times (15, 30 and 45 min) and temperatures (40, 60 and 80 °C) were used. The samples were weighed before and after heating in to measure moisture loss, then the samples were transferred to the screw press at the optimum parameters that were determined in the screw press. Three replicates were made for the experiment. The energy consumption for heating in the ultrasonic and the power for each sample were measured and oil yield was recorded.

### Oil quality measurements

Fatty acid was measured using of gas chromatography (GC) technique according to Azadmard-Damirchi et al.^18^. The fatty acid composition was determined by the transmethylation of the fatty chains to fatty acid methyl esters (FAMEs) according to the modified method by Ref.^24^. The oil samples of 0.2 g were mixed with 6 mL of methanolic sodium hydroxide solution. The mixture is refluxed for 10 min and then, adding HCl (30 mL) and 20 mL methanol and refluxed for another 10 min, then adding 10 mL hexane, and refluxed for another 2 min, and then left to cool. Finally, adding distilled water (10 mL) and poured into separating tunnel. The upper layer is collected and dried with calcium chloride. The FAMEs were separated with an HP 6890 plus gas chromatography (Hewlett Packard, USA), using a capillary column Supelco™ SP-2380 (60 m × 0.25 mm × 0.20 μm), (Sigma-Aldrich, USA), Detector (FID) and the injector and detector temperature was 250 °C. The column temperature was 140 °C (held for 5 min) and rose to 240 °C, at rate of 4 °C/min, and held at 240 °C for 10 min. The carrier gas was helium at flow rate 1.2 mL min^–1^. Sample volume was 1µL (in *n-*hexane) and injected through a split injector at splitting ratio of 100:20. FAMEs were identified by comparing their relative and absolute retention times to those authentic standards of FAMEs (Supelco™ 37component FAME mix). The fatty acid composition was reported as a relative percentage of the total peak area^24^.

Saponification number, Acid value and molecular weight of oil were determined according to Ref.^25^. The acid value of oil was determined by titrating of solution of oil in diethyl ether with alcoholic solution of sodium or potassium hydroxide. Each 1 g of oil is expressed by the amount of KOH which is used to neutralize the oil.

### Transesterification of Jatropha oil

Biodiesel was produced from Jatropha oil according to Gad and Abu Hashish^26^ method. The free fatty acid (FFA) percentage is determined by the Acid value which is 1.382% if is larger than 1% we must produce biodiesel from Jatropha oil by a two-stage transesterification cycle. The biodiesel yield was calculated according to Ibrahim et al.^27^ as shown in Table 1. Also, the energy required to produce biodiesel was measured by a device an energy meter.

**Table 1:** Conditions of Jatropha biodiesel.

Time (min)	Catalyst	Catalyst concentration (%wt)	Alcohol	Molar ratio alcohol: oil	Speed (RPM)	Temperature (°C)
90 min	Sulfuric acid	1%	Ethanol	20%	500–600	65
90 min	NaOH	1%	Methanol	20%	500–600	65

All experimental protocols were approved by Benha university research committee and all methods used in this study were carried out according to the International, National and Benha University guidelines regulations.

### Statistical analysis

The data were subjected to analysis using statistical package SPSS version 21 in which one way ANOVA and Duncan Multiple Range Test (DMRT) were performed at significance level of (p < 0.05) at 95% confidence limit to know the significant differences between the treatment means for different parameters.

## Results and discussion

### Effect of extraction temperature and screw speed on oil extraction yield, extraction time and extraction time for Jatropha seeds

Table 2 shows the effect of extraction temperature (60, 80, 100 and 120 °C) and screw speed (60, 90 and 120 rpm) on oil extraction yield, extraction energy consumption and extraction time for Jatropha seeds. The results indicate that the oil yield increases with increasing extraction temperature and decreasing with increasing screw speed. It could be seen that the oil extraction yield significantly increased from 17.41 to 25.10 (by 30.64%), 15.74 to 23.51 (by 33.05%) and 11.85 to 20.66 (by 42.87%) % when the extraction temperature increased from 60 to 120 °C, respectively, at 60, 90 and 120 rpm. The results also indicate that the highest value of decreasing of oil extraction yield (25.10%) was found for 120 °C extraction temperature. This was may be due to that with higher temperatures, the extraction process of oil is easier treatment^15^.

**Table 2:** Effect of extraction temperature and screw speed on oil yield, extraction energy consumption and extraction time of Jatropha seeds.

Extraction temperature, ℃	Screw speed, rpm	Oil yield, %	Extraction energy consumption, W.h	Extraction time, min
60	60	17.41^b^	12.00^e^	3.70^g^
	90	15.74^b^	9.80^c^	2.65^d^
	120	11.85^a^	8.70^ab^	2.13^b^
80	60	22.00^d^	11.50^e^	3.25^ef^
	90	18.31^bc^	9.50^bc^	2.42^bc^
	120	16.43^b^	8.45^a^	1.75^a^
100	60	23.89^e^	11.00^d^	3.12^e^
	90	20.1^cd^	9.20^b^	2.28^b^
	120	18.83^c^	8.25^a^	1.67^a^
120	60	25.1^e^	10.50^d^	3.00^e^
	90	23.51^de^	8.80^b^	2.20^b^
	120	20.66^d^	8.00^a^	1.60^a^

Means on the same column with different superscripts are significantly different (p < 0.05).

The results indicate that the maximum Jatropha oil production is about 25.1% by the screw press at an engine speed of 60 rpm and preheating temperature of 120 °C. These results agreed with those obtained by Raja et al.^28^. This is due to the oil viscosity decreases with increasing extraction temperature, therefore, the oil exit from the cell easy. However, in these extraction conditions, much time is consumed with higher energy. The optimum yield of oil (23.51%) was obtained at temperatures in the range of 120 °C at speed of 90 rpm, the yield is slightly lower but the energy is much lower than are as will. Although the choice of these conditions reduced the production of the oil yield by 46.92%, but it saved in energy by a percentage of 37.93% and reduced the extraction time by 73.44% compared to the conditions of the highest oil production.

Multiple regression analysis was carried out to obtain a relationship between the oil yield as dependent variable and different both of extraction temperature and screw speed as independent variables. The best fit for this relationship is presented in the following equation:

1 $$ Y = 15.39 + 0.13T - 0.09S{\text{ R}}^{{2}} = 0.95 $$

where Y is the oil yield, %, T is the extraction temperature, °C, S is the screw speed, rpm.

This equation could be applied in the range of 60 to 120 °C of extraction temperature and from 60 to 120 rpm of screw speed.

Regarding, the effect of screw press conditions on extraction energy consumption for Jatropha seeds. The results indicate that the extraction energy consumption decreases with increasing screw temperature and screw speed. It could be seen that the extraction energy consumption significantly decreased from 12.00 to 10.50 (by 12.50%), 9.80 to 8.80 (by 10.20%) and 8.70 to 8.00 (by 8.05%) W.h when the screw temperature increased from 60 to 120 °C, respectively, at 60, 90 and 120 rpm. The results also indicate that the highest value of extraction energy (12.00 W.h) was found of 60 °C extraction temperature and 60 rpm screw speed. These results agreed with those obtained by Ibrahim et al.^18^ whose found lower extraction temperature and screw speed increase energy consumed. While, the energy consumed in extracting oil was as low as 8.0 W.h at 120 °C and 120 rpm, for extraction temperature and screw speed, respectively. These results are in agreement with those obtained by Ofori-Boateng et al.^29^.

Multiple regression analysis was carried out to obtain a relationship between the extraction energy consumption as dependent variable and different both of extraction temperature and screw speed as independent variables. The best fit for this relationship is presented in the following equation:-

2 $$ EC = 15.582 - 0.018T - 0.048S{\text{ R}}^{{2}} = 0.95 \, $$

where Y is the extraction energy consumption, W.h.

This equation could be applied in the range of 60 to 120 °C of extraction temperature and from 60 to 120 rpm of screw speed.

Considering the effect of screw press conditions on extraction time for Jatropha seeds, data obtained in Table 2 showed clearly that, the extraction time decreases with increasing screw temperature and screw speed. It could be seen that the extraction time significantly decreased from 3.70 to 3.00 (by 18.92%), 2.65 to 2.20 (by 16.98%) and 2.13 to 1.60 (by 24.88%) min when the screw temperature increased from 60 to 120 °C, respectively, at 60, 90 and 120 rpm. The results also indicate that the highest value of extraction time (3.7 min) was found of 60 °C extraction temperature and 60 rpm screw speed. These results agreed with those obtained by Ibrahim et al.^18^ whose found lower extraction temperature and screw speed increase extraction time. While, the lowest value of extraction time (1.6 min) was found of 120 °C extraction temperature and 120 rpm screw speed. These results agreed with those obtained by Ofori-Boateng et al.^29^.

Multiple regression analysis was carried out to obtain a relationship between the extraction time as dependent variable and different both of extraction temperature and screw speed as independent variables. The best fit for this relationship is presented in the following equation:-

3 $$ Et = 5.51 - 0.009T - 0.025S{\text{ R}}^{{2}} = 0.97 $$

where Et is the extraction time, min.

This equation could be applied in the range of 60 to 120 °C of extraction temperature and from 60 to 120 rpm of screw speed.

The results also indicate that the optimum condition of screw press for extracted Jatropha seeds were 120 °C extraction temperature and 90 rpm screw speed. It could be seen that the extraction oil yield, extraction energy consumption and extraction time were 23.51%, 8.8 W.h and 2.2 min, respectively.

### Pretreatment conditions

#### Effect of pretreatment conditions (microwave and ultrasonic) on oil extraction yield

##### Microwave treatment

**Figure 3:**
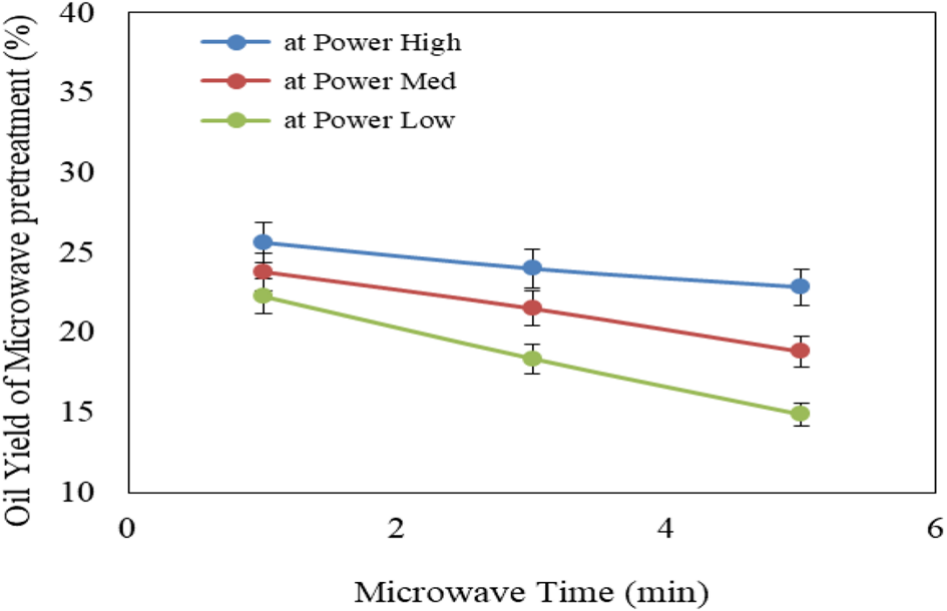
Effect of microwave pretreatment conditions on oil extraction yield from Jatropha seeds.

Figure 3 shows the effect of microwave power levels (low, medium and high) and operating times (1, 3 and 5 min) on oil extraction yield from Jatropha seeds. The results indicate that the oil extraction yield decreases with increasing operating time for low, medium and high microwave power levels. It could be seen that the oil extraction yield significantly decreased from 25.63 to 22.84, 23.8 to 18.8 and 22.26 to 14.87% when the operating time increased from 1 to 5 min, respectively for low, medium and high microwave power levels. This is due to the high microwave power level causes evaporation of oil moisture from the cell, which leads to a decrease of oil yield. The results also indicate that the extraction oil at 120 °C extraction temperature recorded the highest value of oil extraction yield (25.63%) when adjusting on 1 min operating time for the low level of power of microwave treatment. The oil extraction yield was 23.51% at the same condition (120 °C extraction temperature and 90 rpm screw speed). These results are in agreement with those obtained by Khater et al.^30^.

##### Ultrasonic treatment

**Figure 4:**
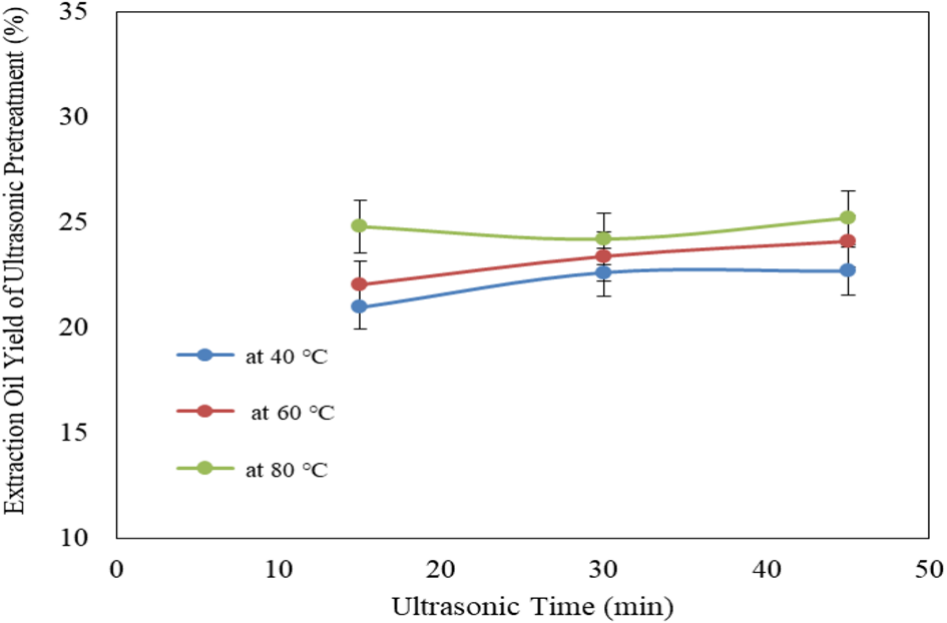
Effect of ultrasonic pretreatment conditions on oil extraction yield from Jatropha seeds.

Figure 4 shows the effect of ultrasonic temperatures (40, 60 and 80 °C) and operating times (15, 30 and 45 min) on oil extraction yield from Jatropha seeds. The results indicate that the oil extraction yield were 20.96, 22.62 and 22.68, 22.04, 23.39 and 24.1and 24.8, 24.2 and 25.2% for 40, 60 and 80 °C ultrasonic temperatures, respectively at 15, 30 and 45 min operating time. The results indicate that the oil extraction yield increases with increasing ultrasonic temperature and operating time. It could be seen that the oil extraction yield significantly increased from 20.96 to 24.80, 22.62 to 24.20 and 22.68 to 25.20% when the ultrasonic temperature increased from 40 to 80 °C, respectively for 15, 30 and 45 min operating time. The results also indicate that the highest value of oil extraction yield (25.2%) was found of 45 min operating time and 80 °C ultrasonic temperature.

Finally, the results also indicate that the oil extraction yield from Jatropha seeds by using microwave pretreatment was lower than those of ultrasonic pretreatment for medium and high power levels and medium and high temperature, while the oil extraction yield from Jatropha by using microwave pretreatment was higher than those of ultrasonic pretreatment for low power levels and low temperature. It could be seen that the oil extraction yield were 24.157, 21.37 and 18.5% for low, medium and high microwave power levels, respectively, but they were 22.086, 23.18 and 24.73% for 40, 60 and 80 °C ultrasonic temperature, respectively. Also, the oil extraction yield form Jatropha seeds by using microwave pretreatment were higher than those of ultrasonic pretreatment for low operating time, while the oil extraction yield for extracted Jatropha seeds from Jatropha seeds by using microwave pretreatment was lower than those of ultrasonic pretreatment for medium and high operating time. It could be seen that the oil extraction yield were 23.897, 21.29 and 18.84% for 1, 3 and 5 min microwave operating time, respectively, but they were 22.6, 23.43 and 23.99% for 15, 30 and 45 min ultrasonic operating time, respectively.

#### Effect of pretreatment conditions (microwave and ultrasonic) on extraction energy consumption

**Figure 5:**
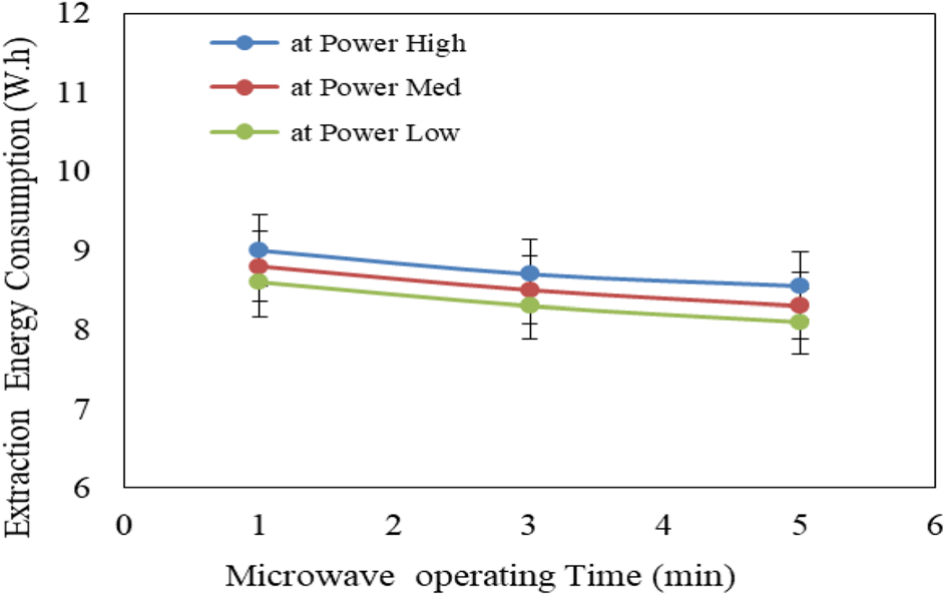
Effect of microwave pretreatment conditions on extraction energy consumption from Jatropha seeds.

Figure 5 shows the effect of microwave power levels (low, medium and high) and operating time (1, 3 and 5 min) on extraction energy consumption from Jatropha seeds. The results indicate that the extraction energy consumption for Jatropha seeds decreases with increasing microwave power level and operating time. It could be seen that the extraction energy consumption for Jatropha seeds decreased from 9.00 to 8.60, 8.70 to 8.30 and 8.55 to 8.10 W.h when the microwave power levels increased from low to high, respectively at 1, 3 and 5 min operating time. On the other hand, the energy consumed by microwave increases with increasing microwave power level and operating time. It could be seen that the energy consumed by microwave significantly increased from 3.0 to 16.0, 13.0 to 67.0 and 18.0 to 83.0 W.h when the operating time increased from 1 to 5 min, respectively for low, medium and high microwave power levels. The results also indicate that the highest value of extraction energy consumption for Jatropha seeds (9.0 W.h) was found of 1 min operating time and low microwave power level. While, the lowest value of extraction energy consumption for Jatropha seeds (8.1 W.h) was found of 5 min operating time and high microwave power level.

**Figure 6:**
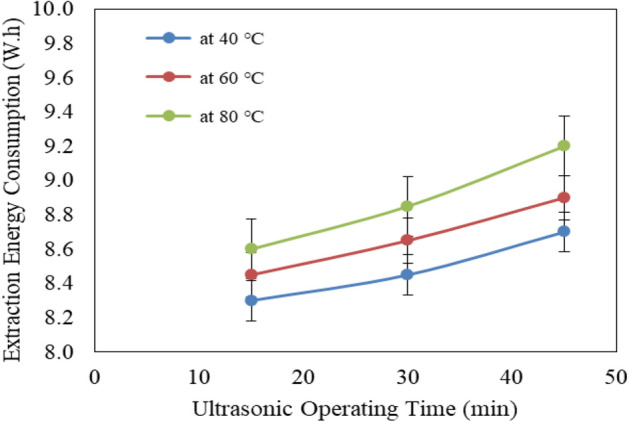
Effect of ultrasonic pretreatment conditions on extraction energy consumption from Jatropha seeds.

Figure 6 shows the effect of ultrasonic temperature (40, 60 and 80 °C) and operating time (15, 30 and 45 min) on extraction energy consumption from Jatropha seeds. The results indicate that the extraction energy consumption for Jatropha seeds increases with increasing ultrasonic temperature and operating time. It could be seen that the extraction energy consumption for Jatropha seeds significantly increased from 8.30 to 8.60, 8.45 to 8.85 and 8.70 to 9.20 W.h when the ultrasonic temperature increased from40 to 80 °C, respectively at 15, 30 and 45 min operating time. While, the energy consumed by ultrasonic increases with increasing ultrasonic temperature and operating time. It could be seen that the energy consumed by ultrasonic increased from 11.00 to 32.00, 15.00 to 34.00 and 18.00 to 38.00 W.h when the operating time increased from 15 to 45 min, respectively for 40, 60 and 80 °C ultrasonic temperature. The results also indicate that the highest value of extraction energy consumption for Jatropha seeds (9.20 W.h) was found of 45 min operating time and 80 °C ultrasonic temperature. While, the lowest value of extraction energy consumption for Jatropha seeds (8.30 W.h) was found of 15 min operating time and 40 °C ultrasonic temperature.

Finally, using MWAV treatments recorded lower values of energy consumption at medium and high level of power compared to ultrasonic treatments. Meanwhile, low level of microwave power recorded higher energy consumption compared to ultrasonic. It could be seen that the extraction energy consumption for Jatropha seeds were 8.75, 8.53 and 8.33 W.h for low, medium and high microwave power levels, respectively, but they were 8.48, 8.67 and 8.88 W.h for 40, 60 and 80 °C ultrasonic temperature, respectively. Also, the extraction energy consumption for Jatropha seeds by using microwave pretreatment was lower than those of ultrasonic pretreatment for medium and high operating time, while the extraction energy consumption by using microwave pretreatment was higher than those of ultrasonic pretreatment for low operating time. It could be seen that the extraction energy consumption were 8.8, 8.5 and 8.32 W.h for 1, 3 and 5 min microwave operating time, respectively, but they were 8.45, 8.65 and 8.933 W.h for 15, 30 and 45 min ultrasonic operating time, respectively.

#### Effect of pretreatment conditions (microwave and ultrasonic) on extraction time

**Figure 7:**
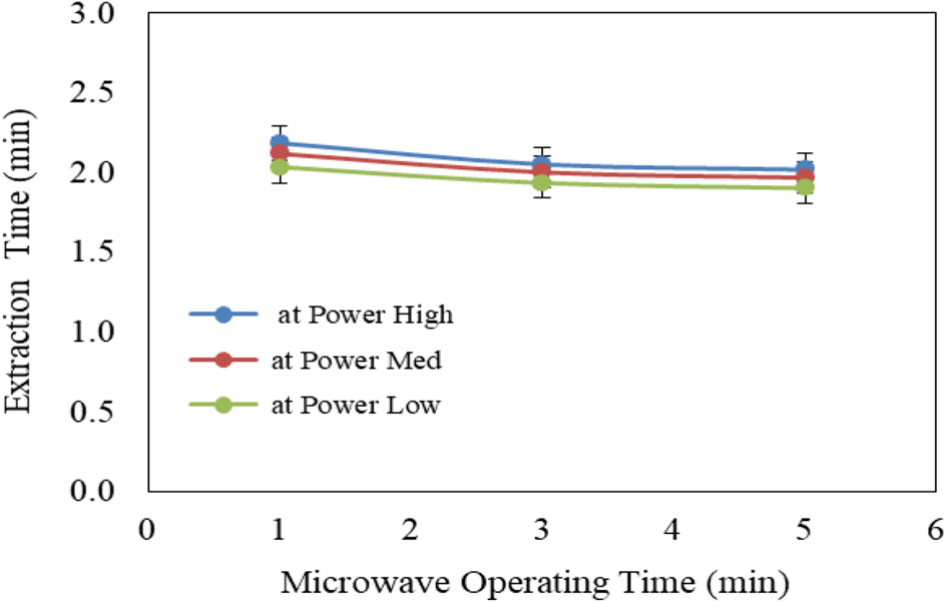
Effect of microwave pretreatment conditions on extraction time from Jatropha seeds.

Figure 7 shows the effect of microwave power levels (low, medium and high) and operating times (1, 3 and 5 min) on extraction time from Jatropha seeds. The results indicate that the extraction time for Jatropha seeds decreases with increasing microwave power level and operating time. It could be seen that the extraction time for Jatropha seeds significantly decreased from 2.18 to 2.017, 2.12 to 1.97 and 2.03 to 1.90 min when the operating time increased from 1 to 5 min, respectively for low, medium and high microwave power levels. The results also indicate that the highest value of extraction time (2.18 min) was found at 1 min operating time and low microwave power level. While, the lowest value of extraction time (1.90 min) was found at 5 min operating time and high microwave power level.

**Figure 8:**
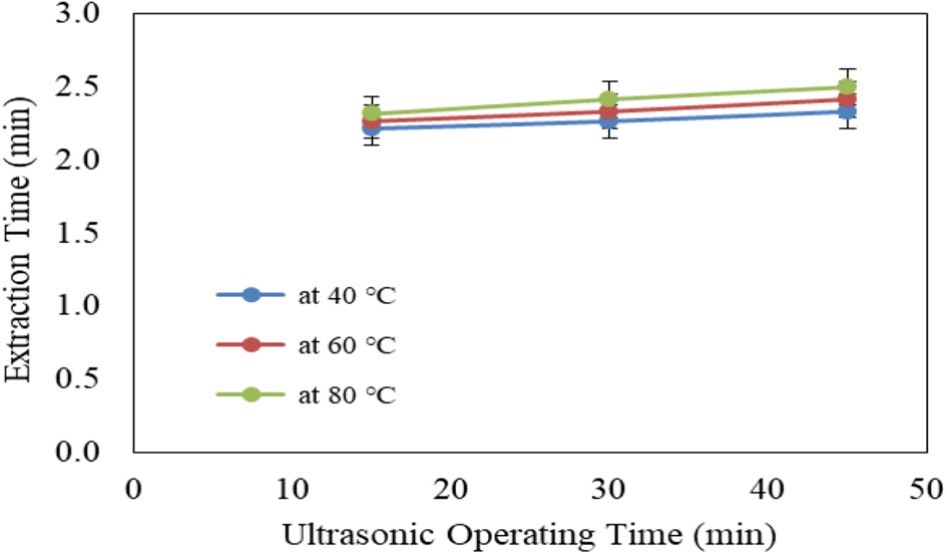
Effect of ultrasonic pretreatment conditions on extraction time from Jatropha seeds.

Figure 8 shows the effect of ultrasonic temperature (40, 60 and 80 °C) and operating time (15, 30 and 45 min) on extraction time from Jatropha seeds. The results indicate that the extraction time for Jatropha seeds increases with increasing ultrasonic temperature and operating time. It could be seen that the extraction time for Jatropha seeds significantly increased from 2.22 to 2.33, 2.27 to 2.42 and 2.32 to 2.50 min when the operating time increased from 15 to 45 min, respectively for 40, 60 and 80 °C ultrasonic temperature. The results also indicate that the highest value of extraction time (2.50 min) was found of 45 min operating time and 80 °C ultrasonic temperature. While, the lowest value of extraction time (2.22 min) was found of 15 min operating time and 40 °C ultrasonic temperature.

### Chemical characteristics of Jatropha oil as affect by study treatments

Table 3 shows the effect of pretreatment (microwave and ultrasonic) of Jatropha seeds on the chemical analysis compared to without pretreatment on the same conditions (120 °C extraction temperature and 90 rpm screw speed). The results indicate that, using of microwave for pretreatment of Jatropha seeds was enhanced contents of oil yield properties more than those of ultrasonic pretreatment for Jatropha seeds and without pretreatment. On the other hand, using of ultrasonic for pretreatment of Jatropha seeds was enhanced quality of oil properties more than those of microwave pretreatment for Jatropha seeds and without pretreatment. It could be seen that the Palmitic acid, Stearic acid, Oleic acid, Linolenic acid and α Linolenic acid were 15.25, 17.61 and 15.92, 2.33, 1.86 and 1.07, 5.65, 5.82 and 5.13, 36.53, 35.22 and 36.35 and 40.24, 39.49 and 41.53% for non-treatment, microwave pretreatment and ultrasonic pretreatment, respectively. These results agreed with those obtained by Ogunniyi^31^ and Conceicao et al.^32^ whose found that the castor oil is presents in minor amount that includes Stearic acid (1%), Linoleic acid (4.2%), Linolenic acid (0.3%), Dihydroxystearic acid (0.7%),Oleic acid (3.0%), Palmitic acid (1%), and Eicosanoic acid (0.3%)^15^.

**Table 3:** Chemical analysis of treated and non-treated Jatropha seeds.

Item	Without	Pretreatment	
	Pretreatment	Microwave	Ultrasonic
Palmitic acid (C16:0)	15.25^a^	17.61^b^	15.92^a^
Palmitoleic acid (C16:1), n9	2.33^c^	1.86^b^	1.07^a^
Stearic acid (C18:0)	5.65^b^	5.82^b^	5.13^a^
Oleic acid (C18:1n9c)	36.53^b^	35.22^a^	36.35^a^
Linoleic acid (C18:2n6c)	40.24^b^	39.49^a^	41.53^c^
Acid value (mg/g)	2.76^a^	3.34^b^	2.88^b^
Saponification value (mg/g)	191.7^a^	192.8^a^	191.4^a^
Molecular weight (g/mol)	911.57^a^	910.18^a^	912.00^a^

Means on the same row with different superscripts are significantly different (p < 0.05).

The results indicate that the acid values were 2.76, 3.34 and 2.88% for non-treatment, microwave pretreatment and ultrasonic pretreatment, respectively. Acid value is one of the important indicators of oil quality^16^. Omari et al.^33^ suggested that the high acid value of Jatropha oil may be due to the delay in seed extraction which influenced the lipase enzyme to hydrolyze the triglycerides into free fatty acid^34^.

Saponification number values were 191.7, 192.8 and 191.4 for non-treatment, microwave pretreatment and ultrasonic pretreatment, respectively. These results agreed with those obtained by Attia et al.^3^ and Yeboah et al.^34^. Also, the physicochemical properties of oil such as low acid value and free fatty acid percentage, high saponification value acid indicate that castor oil has good oil quality.

The molecular weight (MW) of oil value were 911.57, 910.18 and 912.00 for non-treatment, microwave pretreatment and ultrasonic pretreatment, respectively. The raw materials are converted into biodiesel through a chemical reaction involving alcohol and a catalyst. The specification of the molecular weight of crop oil is important for the biodiesel production process because the determination of the quantity of reactants is calculated according to the molecular weight of castor oil^35^. The results indicate that the lowest and the best of the molecular weight (MW) of oil value ultrasonic pretreatment then without pretreatment then microwave pretreatment.

### Biodiesel production

The yield of biodiesel from 1 kg of oil was 0.916 kg. The efficiency of biodiesel production from Jatropha oil was 91.64%. The energy consumed from the magnetic stirrer and washing biodiesel by using boiler were 875 and 1177.5 W.h per kg oil, respectively. The total energy consumed to produce biodiesel from Jatropha oil was 2052.5 W.h per kg oil.

### Physical and chemical properties of biodiesel produced from Jatropha oil

Table 4 shows the physical and chemical properties of biodiesel production from Jatropha oil compared to diesel oil. The results indicate that the density was 918.6 and 887.1 kg m^–3^ for Jatropha oil and Jatropha biodiesel, respectively, compared to 829 kg m^–3^ of diesel density. The kinematic viscosity was 34.33 and 5.69 m^2^ s^–1^ for Jatropha oil and Jatropha biodiesel, respectively, compared to 1.20 m^2^ s^–1^ of diesel kinematic viscosity. The dynamic viscosity was 6.73 and 3.25 mPa.s for Jatropha oil and Jatropha biodiesel, respectively, compared to 1.43 mPa.s of diesel dynamic viscosity. The flash point was 146 and 121 °C for Jatropha oil and Jatropha biodiesel, respectively, compared to 73.55 °C of diesel flash point.

**Table 4:** Properties of biodiesel production from Jatropha oil compared to diesel fuel.

Property	Diesel	Jatropha oil	Jatropha biodiesel
Density at 15.56 °C, kg m^–3^	829^a^	918.6^c^	887.1^b^
Kinematic viscosity at 40 °C,.mm^2^ s^–1^	1.2^a^	34.328^b^	5.6965^b^
Dynamic viscosity, mPa.s	1.43^a^	6.726^c^	3.2526^b^
Flash point, °C	73.55^a^	146^c^	121^b^
Heating value, kJ kg^–1^	42,000^a^	45,500^b^	45,000^b^
Cloud point, °C	− 6^a^	− 1^b^	0^b^
Cetane number	41^a^	39.12^a^	73^b^

Means on the same row with different superscripts are significantly different (p < 0.05).

The results also indicate that the heating value was 45,500 and 45,000 kJ kg^–1^ for Jatropha oil and Jatropha biodiesel, respectively, compared to 42,000 kJ kg^–1^ of diesel heating value. The cloud point was − 1 and 0 °C for Jatropha oil and Jatropha biodiesel, respectively, compared to − 6 °C of diesel cloud point. The cetane number was 39.12 and 73 for Jatropha oil and Jatropha biodiesel, respectively, compared to 41 of diesel cetane number. Cetane number indicates the tendency to knock. Cetane number is a measure of the combustion quality of a fuel in diesel engine and is related to the volatility of the fuel and ignition delay time. Ignition quality and cetane number affect engine performance, cold starting, warm up, and engine combustion roughness. The higher cetane number led to shorter ignition delay and engine performance reduction. Cetane number can be used without problems in fuel atomization and spray. Biodiesel burns with higher combustion efficiency. Cloud point demonstrates the biodiesel’s low-temperature operation. Long-chain saturated fatty acids have a significant impact on the cold flow characteristics of biodiesel. Biodiesel can be operated efficiently in cold conditions. Properties of biodiesel produced from Jatropha oil are compared with diesel. Better physical and chemical properties are for Jatropha biodiesel compared with diesel.

## Conclusions

This work was seeking the optimum conditions of oil extraction process from Jatropha seeds and how to enhance the extraction yield of oil by using microwave and ultrasonic pre-treatments. For the results of this study, the most important results concluded as follows: the optimum conditions to obtain highest oil yield (23.51%) were 120 °C extraction temperature for 2.2 min at 90 rpm screw speed. Using microwave pre-treatment at low power level gave the highest oil yield (24.16%), while using ultrasonic treatment at 80 °C gave the highest oil yield (24.73%). The energy requirement for extraction ranged from 2.3 to 8.8 W.h depending on the treatments under study. The efficiency of biodiesel production from Jatropha oil was 91.64%. The total energy consumed to produce biodiesel from Jatropha oil was 821W.h. The pretreatment improved the physical and chemical properties of the biodiesel production from Jatropha seeds.

## Data Availability

The datasets used and/or analyzed during the current study available from the corresponding author on reasonable request.
